# Supplementary material for: Identification of photoperiod-induced specific miRNAs in the adrenal glands of Sunite sheep (Ovis aries)
Source: Front Vet Sci. 2022 Jul 22;9:888207. doi: 10.3389/fvets.2022.888207 (PMC9354845; doi:10.3389/fvets.2022.888207)
Supplement: Supplementary file 1 [file Data_Sheet_1.ZIP › Supplementary file/miRDeep2 file.zip!/miRDeep2 file/Novel_Structure/SPLP42A2/Novel_515.pdf]

Provisional ID	:	NC_019483.2_41453
Score total	:	29.4
Score for star read(s)	:	3.9
Score for read counts	:	22
Score for mfe	:	1.9
Score for randfold	:	1.6
Score for cons. seed	:	
Total read count	:	55
Mature read count	:	50
Loop read count	:	0
Star read count	:	5

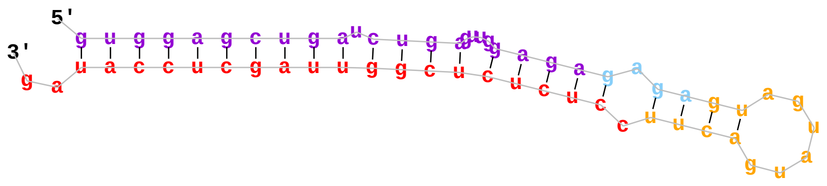

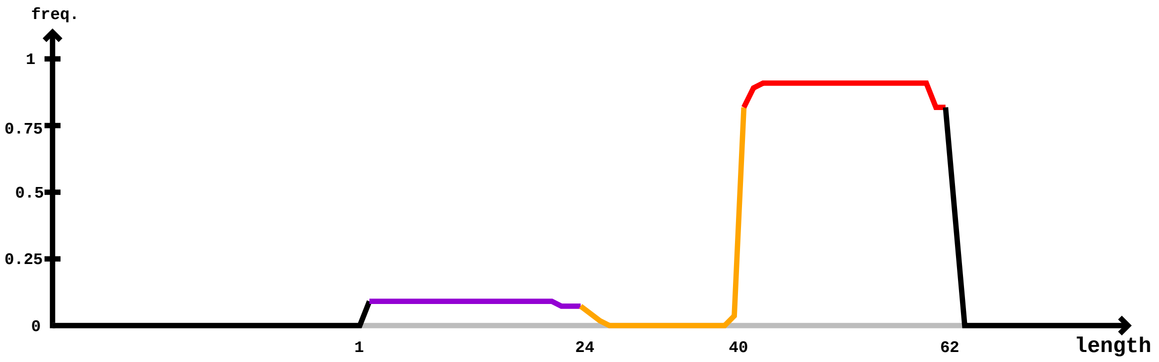

Star Mature

```
5'- uuuacucaguucacugaccgauugugccaguguggagcugaucugaguuggagagagaguaguaugacuuccucucucgguuagcuccauagauggcagaucagccuucu   -3'   obs
    uuuacucaguucacugaccgauugugccaguguggagcugaucugaguuggagagagaguaguaugacuuccucucucgguuagcuccauagauggcagaucagccuucu         exp
    .....((((....))))..((((.(((((.(((((((((((.((((....(((((.((((......)))).))))))))))))))))))))..)))))))))........   reads   mm   sample
    ...............................guggagcugaucugaguugg...........................................................   1       0    seq
    ...............................guggagcugaucugaguuggaga........................................................   3       0    seq
    ...............................guggagcugaucugaguuggagaga......................................................   1       0    seq
    .....................................................................uccucucucgguuagcuccauag..................   2       0    seq
    ......................................................................ccucucucgguuagcuccau....................   4       0    seq
    ......................................................................ccucucucgguuagcuccauag..................   31      0    seq
    ......................................................................ccucucucgguuagcuccauaga.................   8       0    seq
    .......................................................................cucucucgguuagcuccau....................   1       0    seq
    .......................................................................cucucucgguuagcuccauag..................   1       0    seq
    .......................................................................cucucucgguuagcuccauaga.................   2       0    seq
    ........................................................................ucucucgguuagcuccauag..................   1       0    seq
```
